# Are weight status and weight perception associated with academic performance among youth?

**DOI:** 10.1186/s40337-020-00329-w

**Published:** 2020-10-26

**Authors:** Maram Livermore, Markus J. Duncan, Scott T. Leatherdale, Karen A. Patte

**Affiliations:** 1 Department of Health Sciences, Brock University, Faculty of Applied Health Sciences, Niagara Region, 1812 Sir Isaac Brock Way, St. Catharines, ON L2S 3A1 Canada; 2 School of Public Health and Health Systems, University of Waterloo, 200 University Avenue, Waterloo, Ontario N2L 3G1 Canada

**Keywords:** Obesity, Overweight, Academic achievement, Weight perception, Education, Youth

## Abstract

**Background:**

Emerging evidence suggests perceptions of being overweight account for many of the psychosocial consequences commonly associated with obesity. Previous research suggests an obesity achievement gap, yet limited research has explored weight perception in association with academic performance. Moreover, underweight perceptions have typically been excluded from research. The current study examined how BMI classification and weight perception relate to academic performance in a large cohort of youth.

**Methods:**

We used cross-sectional survey data from 61,866 grade 9–12 students attending the 122 Canadian schools that participated in Year 6 (2017/2018) of the COMPASS study. Mixed effect regression models were used to examine associations between students’ BMI classification and weight perceptions and their math and English/French course grades. All models were stratified by sex and adjusted for sociodemographic covariates and school clustering.

**Results:**

For English/French grades, males and females with overweight or underweight perceptions were less likely to achieve higher grades than their peers with perceptions of being at “about the right weight”, controlling for BMI and covariates. For math grades, females with overweight perceptions, and all students with underweight perceptions, were less likely to achieve higher grades than their peers with “about the right weight” perceptions. All students with BMIs in the obesity range were less likely to report grades of 60% or higher than their peers with “normal-weight” BMIs, controlling for weight perception and covariates. Overweight BMIs were predictive of lower achievement in females for English/French grades, and in males for math grades, relative to “normal-weight” BMIs. Results for students that did not respond to the weight and weight perception items resembled those for obesity BMI and overweight/underweight perceptions, respectively.

**Conclusions:**

Overall, this study demonstrates that an obesity achievement gap remains when controlling for students’ perceptions of their weight, and that both underweight and overweight perceptions predict lower academic performance, regardless of BMI classification. Results suggest barriers to academic success exist among youth with larger body sizes, and those with perceptions of deviating from “about the right weight”.

## Plain language summary

An obesity achievement gap has been suggested as an early contributor to later socioeconomic disparities found by weight status. To date, limited research has examined how sociocultural weight norms contribute to academic performance. Emerging evidence suggests weight perception—individuals’ subjective appraisal of their body weight—accounts for many of the psychosocial consequences commonly associated with obesity. We sought to determine if body weight and weight perceptions predicted grades in a large sample of Canadian secondary school students. It was hypothesized that perceptions of being at “about the right weight” would provide a protective effect. Overall, this study demonstrates that an obesity achievement gap remains when controlling for students’ perceptions of their weight, and that weight perceptions—both underweight and overweight—predict academic performance, regardless of BMI classification. Results contribute to a body of research that encourages the consideration of both overweight and underweight perceptions and their potential impact on adolescent emotional and physical health. Further research is needed to determine the mechanisms underlying these relationships, in order to remove barriers to academic success among youth with larger body sizes, and those with perceptions of deviating from “about the right weight”.

## Introduction

About 35% of Canadian children and adolescents are at risk of having overweight or obesity [1]. Numerous studies have shown that childhood obesity is associated with various physical health concerns [2]. Additionally, larger-bodied adolescents are at increased risk of adverse psychosocial outcomes [3]. Strauss and Pollack reported that children and adolescents face many challenges but “few problems in childhood have as significant an impact on emotional development as being overweight” (p.747) [4]. In fact, children with overweight or obesity report lower quality of life scores than children diagnosed with cancer [5]. Previous research also indicates the presence of an obesity achievement gap for children and adolescents [6, 7]. More specifically, some evidence suggests students with obesity have poorer academic achievement, more absenteeism, higher dropout rates [6, 8], and are less likely to pursue and attain post-secondary education [9, 10].

A recent meta-analysis by He et al. included 60 studies of weight status and academic performance and found a pooled correlation (*r* = −.111) between higher Body Mass Index (BMI) and lower grades [11]. The researchers concluded that the relationship between weight status and academic achievement was moderated by geographical region, with lower grades more likely to be associated with high BMI in North America than other cultures, possibly due to differing weight norms and given the stigma associated with overweight and obesity. Emerging evidence suggests weight perception—individuals’ subjective appraisal of their body weight—accounts for many of the psychosocial consequences commonly associated with obesity [12–14]. That is, the perception of being overweight, rather than body weight itself, may account for risks of lower self-concept and poor mental health. One of the potential factors linking obesity and the perception of overweight to adverse outcomes is the experience of stigma and bias. An individual may need to perceive themselves as overweight, in order to internalize the bias associated with overweight/obesity. To date, the relationship between self-perceptions of weight and academic achievement has been largely overlooked. To our knowledge, only one study has examined weight perception as a predictor of academic achievement [15]. Among US adolescents (ages 14 to 17) participating in the 2003 Youth Risk Behavior Study, Florin, Shultz, and Stettler found that perceived overweight status was associated with lower grades, regardless of BMI classification, and that obesity was no longer associated with grades when controlling for weight perception [15]. Replication is necessary, particularly in more recent samples, given potential shifts in social weight norms (e.g., related to obesity prevalence, body positive movements, and anti-obesity stigma and weight bias efforts). Furthermore, consideration of underweight perceptions in addition to overweight, and exploration of sex or gender differences, are warranted. Related to thinness and muscularly sociocultural body ideals, girls/women are more likely to report perceptions of underweight than boys/men, while boys/men tend to be split between perceptions of underweight and overweight [16]. Regardless of body size, both overweight and underweight perceptions appear detrimental to mental and physical health relative to perceptions of being “about the right weight” [12–14, 16–25]; however, the latter has received relatively limited attention.

Further examination of a potential weight-related achievement gap is critical to inform a learning environment that will enable all youth to thrive. Deterrents to academic achievement in adolescence have critical implications for future career opportunities and successful transitions to adulthood, with school failure and dropout increasing the risk of later unemployment, poverty, lower quality life, criminality, violence, and various health risk behaviors [6, 7, 26]. We sought to determine if larger-bodied students report lower grades in secondary school than their peers with “normal-weight” BMIs. In addition, we explored whether weight perceptions predicted grades, while controlling for weight status. It was hypothesized that perceptions of being at “about the right weight” would provide a protective effect, and any relationship between BMI classification and academic achievement would be reduced when controlling for weight perception.

## Methods

### Design and participants

The COMPASS study is a prospective cohort study designed to collect longitudinal and hierarchical health data from a large sample of grade 9 through 12 students (ages 14–19) enrolled in Canadian secondary schools. Schools and school boards were purposely selected based on whether they permitted active-information passive-consent parental permission protocols, which are critical for collecting robust data among youth [27]. The COMPASS student questionnaire (Cq), a self-report paper-and-pencil survey, is completed once annually by full school samples during one classroom period. All grade 9 through 12 students attending participating schools were eligible to participate and could decline at any time. A full description of recruitment methods [28] and the COMPASS study are available in print [29] and online (www.compass.uwaterloo.ca). The COMPASS study received ethics approval from the University of Waterloo and Brock University Human Research Ethics Committee and all participating school boards.

We used cross-sectional data from Year 6 (2017–2018 school year) of the COMPASS study, which included 66,434 students at 122 secondary schools in Ontario (*n* = 61), British Columbia (*n* = 16), Alberta (*n* = 8) and Quebec (*n* = 37). The overall student response rate in Year 6 was 81.85% of eligible students. Student non-participation primarily resulted from absences or scheduled study-periods during data collection. Participants with missing outcome, sex, or covariate data were removed, leaving a final sample of 61,866 adolescents.

### Measures

#### Weight status

Student weight status was defined by Body Mass Index (BMI; kg/m^2^) classification determined based on student-reported height and weight [30], and the World Health Organization [31] age-and sex-adjusted cut points (underweight, normal weight, overweight, obesity). A previous study found the weight status measure to be reliable, valid, and valuable for use when objective methods are not feasible [30]. Given the prevalence of missing BMI data, and as missing self-reported weight data may not be missing at random [32], two separate categories were created for missing weight status based on which variables were missing to determine BMI classification: missing BMI classification due to weight not being reported, and missing BMI due to missing age, sex, or height.

#### Weight perception

Subjective perception of weight status was determined using the question, “How do you describe your weight?” Response options included: “very underweight”, “slightly underweight”, “about the right weight”, “slightly overweight” and “very overweight”. Responses were collapsed into three categories: underweight, about right, and overweight. In addition, missing weight perception responses were included as a fourth category.

#### Covariates

Participant-reported race/ethnicity (categorized into white and, non-white minority, multiethnic, or other) and school grade (9, 10, 11, 12, other [Secondary I- II in Quebec]) were entered into the model as covariates. Also, student weekly spending/saving money (categorized into $1–$20, $21–$100, >$100, don’t know) was included as an indicator of part-time employment and/or allowance, as proxy for student-level SES in the absence of data on parental income or education data.

#### Academic performance

Academic performance was assessed using student-reported grades. Participants reported their approximate overall mark in their current or most recent math and English (in Ontario, Alberta and BC schools) or French (in Quebec) courses. Grades were dichotomized as ≥60% or < 60% for both math and English/French grade models.

### Statistical analysis

R software [33] was used to conduct frequency descriptive statistics and mixed effect logistic regression models. Separate models explored the association between BMI status and weight perception with math grades and English/French course grades, stratified by sex and controlling for weekly spending money, school grade, and race/ethnicity. Mixed models were used to account for school clustering by adding a random intercept at the school level. No interaction effect between weight status and weight perception was indicated when tested (results not reported).

## Results

### Descriptive statistics

Descriptive statistics for all variables are described in Table 1. In this sample, 49.4% of participants identified as male and 50.6% as female. About two-thirds (66.6%) of the sample identified as white and one-third as non-white, mixed, or other race/ethnicity. In terms of weight status, 5.7, 12.0, 54.7 and 3.6% of the total sample had BMIs in the obesity, overweight, “normal-weight”, and underweight categories, respectively, while 19.6% of students did not report their weight, and the remaining proportion were missing sex, age, or height data to categorize BMI. Additionally, a total of 23.8 and 58.7% students reported overweight and “about the right weight” perceptions respectively, while 1.4% did not respond to the weight perception item. More males reported perceptions of “underweight” than females (21.2% versus 11.1%), while more females reported perceptions of overweight (26.5% versus 20.6%) and “about the right weight” (60.7% versus 56.2%) than males. Just over half of students (51.0–51.3%) reported math and English/French grades above 60%.

**Table 1:** Descriptive statistics for secondary school students in Year 6 (2017/2018) of the COMPASS study (*N* = 61,886)

	Female (*n* = 31,334)	Male (*n* = 30,552)
	% (n)	% (n)
Grade		
9	24.3 (7614)	24 (7332)
10	24.5 (7677)	24.3 (7424)
11	23.3 (7301)	23.1 (7058)
12	14.9 (4669)	15.7 (4797)
Other^a^	13.0 (4073)	12.9 (3941)
Race/ethnicity		
White	66.7 (20889)	66.6 (20355)
Non-white, multietnic, or others	33.3 (10445)	33.4 (10197)
BMI Classifications		
Underweight	1.5 (470)	2.1 (642)
“Normal Weight”	56.8 (17798)	50.6 (15459)
Overweight	10.3 (3227)	13.3 (4063)
Obesity	4.1 (1285)	7.4 (2261)
Missing weight	21.7 (6799)	19.4 (5927)
Missing age, sex, or height	5.6 (1755)	7.2 (2200)
Weight Perception		
Underweight	11.1 (3478)	21.2 (6477)
“About the right weight”	60.7 (19019)	56.2 (17170)
Overweight	26.5 (8304)	20.6 (6294)
Missing	1.7 (533)	2.0 (611)
Weekly Spending Money		
None	14.2 (4449)	17.9 (5469)
$1–$20	25.9 (8116)	24.7 (7546)
$21–$100	25.5 (7990)	22.0 (6721)
>$100	16.9 (5295)	20.4 (6233)
Don’t know	17.5 (5484)	15.0 (4583)
Math Grades		
60–100%	51.0 (15980)	51.2 (15643)
0–59%	49.0 (15354)	48.8 (14909)
English/French Grades		
60–100%	51.3 (16074)	51.1 (15612)
0–59%	48.7 (15260)	48.9 (14940)

^a^Secondary I-II in Quebec schools

*BMI* body mass index

**Fig. 1:**
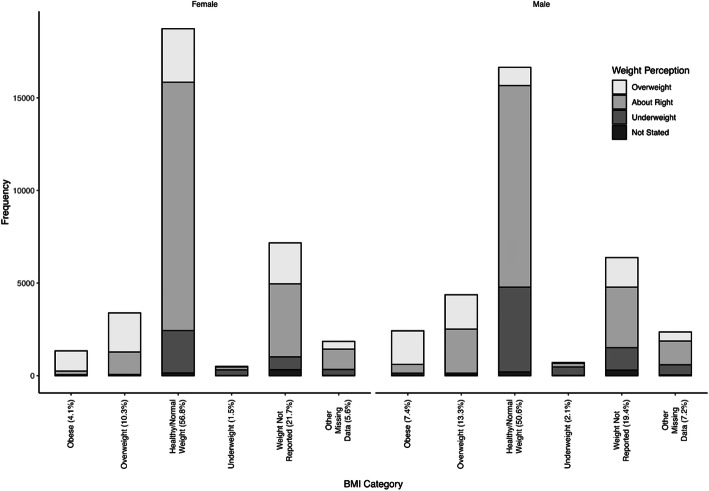
Weight perception and BMI category concordance among female and male secondary school students participating in Year 6 (2017/2018) of the COMPASS study

The concordance between weight perception and BMI category is presented in Fig. 1. The weighted Kappa was 0.392 among females and 0.370 among males. The majority of males (74.9%) and females (81.3%) with BMIs in the obesity category reported perceptions of overweight. Among those with overweight BMIs, most females reported perceptions of overweight (62.2%); whereas more males reported “about right” weight perceptions (54.5%) than overweight perceptions (42.4%). Most males (64.2%) and females (61.5%) in the underweight BMI category reported underweight perceptions. In the “normal weight” BMI category, more males reported underweight perceptions (27.5%) than females (12.2%), while females were more likely to perceive their weight as “about right” (71.6%) or overweight (15.4%) than males (65.4%; 5.9%).

Students with missing BMI data resembled those with “normal weight” BMIs, except a higher proportion reported overweight perceptions. About half of students with missing BMI data reported “about right” weight perceptions (51.1% males; 55.1% females), over a quarter reported overweight perceptions (25.1% males; 30.8% females), and 9.7% of females and 19.0% of males reported underweight perceptions.

### Math course grades

See Table 2 for model results testing BMI classification and weight perception as predictors of math grades, after stratifying by sex and controlling for covariates. In males only, having over $100 a week available for spending or saving and identifying as nonwhite, mixed, or other race/ethnicity were associated with lower odds of reporting math grades over 60%, relative to males without any weekly spending money and of white race/ethnicity, respectively. Students with BMIs in the obesity range were less likely to report a math grade above 60% compared to those with BMIs considered “normal weight”. In males only, overweight BMIs were associated with a lower likelihood of report a math grade above 60% compared to those with “normal-weight” BMIs. No effect resulted for underweight BMI relative to “normal-weight” BMI. In females only, weight perceptions of overweight predicted a lower likelihood of reporting math grades above 60% when compared to perceptions of being “about the right weight”, controlling for BMI classification and covariates. In both males and females, perceptions of underweight were associated with lower likelihood of higher math grades than perceptions of being “about the right weight”. Student with missing BMI data either due to not reporting weight or missing height, age or sex data were more likely to have lower math grades when compared to those in the “normal-weight” BMI category. Similarly, students with missing weight perception data were more likely to report lower math grades when compared to those with “about the right weight” perceptions.

**Table 2:** Mixed-effect model testing BMI category and weight perception as predictors of Math grades ≥60% among youth

	Females (*N* = 31,334)		Males (*N* = 30,552)	
	AOR	95% CI	AOR	95% CI
BMI Category (Reference: “Normal weight”)				
Obesity	0.70**	**0.59–0.82**	0.62**	**0.55–0.71**
Overweight	0.90	0.80–1.02	0.87*	**0.79–0.97**
Underweight	0.93	0.70–1.25	0.85	0.68–1.07
Missing weight data	0.56**	**0.51–0.61**	0.62**	**0.57–0.68**
Missing sex, age, or height data	0.64**	**0.55–0.74**	0.65**	**0.57–0.74**
Weight Perception (Reference: “About the right weight”)				
Overweight	0.82**	**0.75–0.89**	0.91	0.83–1.00
Underweight	0.77**	**0.69–0.86**	0.90*	**0.82–0.97**
Missing weight perception	0.64**	**0.50–0.83**	0.59**	**0.47–0.73**
Race/ethnicity (Reference: White)				
Nonwhite, multiethnic, or other	0.92	0.85–1.00	0.87**	**0.81–0.94**
Grade (Reference: 9)				
10	0.82**	**0.74–0.90**	0.80**	**0.73–0.88**
11	0.96	0.87–1.07	0.83**	**0.75–0.91**
12	0.91	0.81–1.02	0.88	0.79–0.99
Other^a^	1.10	0.95–1.26	1.14	0.99–1.31
Weekly Spending Money (Reference: None)				
$1–$20	0.94	0.84–1.05	0.93	0.84–1.02
$21–$100	0.94	0.84–1.05	0.89	0.80–0.99
> $100	0.94	0.83–1.07	0.86*	**0.77–0.95**
Don’t know	1.05	0.93–1.19	1.02	0.91–1.14

Note: Cross-sectional data from Year 6 (2017/2018) of the COMPASS study (*N* = 61,886). The outcome reference category is math grades < 60%. Mixed models were used to account for school clustering by adding a random intercept at the school level

*p < .01

**p < .001

^a^Secondary I-II in Quebec schools

### English/French course grades

Table 3 summarizes the resultant model testing BMI classification, weight perception, and covariates as predictors of English/French grades by sex. Both males and females identifying as nonwhite, mixed, or other race/ethnicity had lower odds of English/French grades above 60% than their counterparts identifying as white. No differences in English/French grades resulted by spending money or grade. Both male and female adolescents with BMIs in the obesity range, and females with overweight BMIs, were less likely to report higher grades than their peers with BMIs considered “normal weight”.

**Table 3:** Mixed-effect model testing BMI category and weight perception as predictors of English/French grades ≥60% among youth

	Females (*N* = 31,334)		Males (*N* = 30,552)	
	AOR	95% CI	AOR	95% CI
BMI Category (Reference: “Normal weight”)				
Obesity	0.51**	**0.41–0.64**	0.63**	**0.55–0.73**
Overweight	0.71**	**0.60–0.85**	0.87	0.77–0.99
Underweight	1.04	0.68–1.59	0.93	0.71–1.21
Missing weight data	0.45**	**0.40–0.50**	0.54**	**0.49–0.60**
Missing sex, age, or height data	0.49**	**0.40–0.59**	0.58**	**0.51–0.66**
Weight Perception (Reference: “About the right weight”)				
Overweight	0.76**	**0.68–0.85**	0.83**	**0.75–0.92**
Underweight	0.59**	**0.51–0.69**	0.87*	**0.79–0.95**
Missing weight perception	0.50**	**0.36–0.68**	0.66**	**0.51–0.84**
Race/ethnicity (Reference: White)				
Nonwhite, multiethnic, or other	0.67**	**0.60–0.75**	0.74**	**0.68–0.80**
Grade (Reference: 9)				
10	0.96	0.83–1.10	0.94	0.85–1.05
11	0.87	0.75–1.00	0.94	0.84–1.05
12	1.07	0.90–1.27	1.06	0.93–1.21
Other^a^	1.19	0.98–1.44	1.12	0.97–1.29
Weekly Spending Money (Reference: None)				
$1–$20	0.88	0.75–1.03	1.08	0.97–1.21
$21–$100	0.95	0.80–1.11	1.05	0.93–1.18
> $100	0.88	0.74–1.06	0.93	0.83–1.05
Don’t know	0.99	0.84–1.18	1.09	0.96–1.23

Note: Cross-sectional data from Year 6 (2017/2018) of the COMPASS study (*N* = 61,886). The outcome reference category is English/French grades < 60%. Mixed models were used to account for school clustering by adding a random intercept at the school level

*p < .01

**p < .001

^a^Secondary I-II in Quebec schools

Both males and females with weight perceptions of overweight and underweight were less likely to report higher grades (above 60%) in their English/French classes when compared to those with perceptions of being at “about the right weight”. Students with missing BMI data due to either not reporting weight, or because of missing height, age, or sex data, had significantly lower odds of reporting English/French grades above 60%, when compared to those with “normal-weight” BMIs. Similarly, students with missing weight perception data were less likely to have English/French grades above 60% when compared to those with “about the right weight” perceptions.

## Discussion

The current study examined whether weight status and student perceptions of their weight were associated with academic grades in selected secondary school courses (math and English/French) in a large population study of Canadian youth. Results support the links between obesity, as determined by BMI, and lower academic performance in both males and females. Overweight BMI classifications were also associated with lower odds of high grades in English/French courses among females, and in math classes among male students, relative to BMIs considered “normal-weight”. Similar to previous research [15], females with overweight perceptions were less likely to achieve higher course grades in math, and both males and females with overweight perceptions had lower odds of grades above 60% in their English/French courses, relative to their peers with perceptions of being at “about the right weight”, controlling for BMI classification and covariates. While no effect was found for underweight relative to “normal-weight” BMI, females and males with perceptions of being underweight had lower odds of high grades in math and English/French courses relative to students with “about the right weight” perceptions. Results for youth with missing BMI or weight perception data resembled those for obesity BMI classifications and perceptions of overweight or underweight, respectively. Overall, this study demonstrates that an obesity achievement gap remains when controlling for students’ perceptions of their weight, and that weight perceptions—both underweight and overweight—predict lower academic performance, regardless of BMI classification. Further research is needed to determine the mechanisms underlying these relationships, in order to remove barriers to academic success among youth with larger body sizes, and those with perceptions of deviating from “about the right weight”.

To the best of our knowledge, only one previous study has examined weight perception as a predictor of academic grades. The current study provides necessary replication and builds on existing literature, by examining these relationships in a more recent and larger sample of youth. In a sample of approximately 11,000 US adolescents participating in the 2003 Youth Risk Behavior Study, perceptions of overweight were found to be a stronger predictor of academic outcomes than BMI, and obesity was no longer a significant predictor of academic performance when accounting for overweight perceptions [15]. Overweight perceptions have been linked to poor mental health, psychosocial distress, and low self-esteem in adolescents [26, 34–36], factors that have also been associated with lower academic achievement [37]. In fact, researchers have reported perceiving oneself as overweight to be a stronger predictor of behavioural issues and mental distress than actual weight status [12, 36, 38]. Similarly, the current results indicate that perceptions of overweight significantly predicted poorer academic outcomes independent of BMI classification, with the exception of math grades in males. However, unlike Florin et al. [15], obesity BMI classifications remained predictive of lower grades when controlling for weight perception.

The current study suggests other factors appear to contribute to the obesity achievement gap, such as parental education, mental health, or external weight bias. Students with obesity are more likely to experience weight-based bullying within the school context [15, 35]. Also, weight bias has been documented in physical education teachers [9, 39–41] and given its pervasiveness across the population [9, 39], may contribute to educators’ perceptions of students’ academic abilities. Several studies have indicated that the association between BMI and academic performance was no longer significant when models adjusted for parental/familial characteristics [42, 43]. For instance, Datar et al. concluded that overweight status is not a causal factor of lower academic performance, as weight-related differences in test scores became insignificant when social and behavioural variables, such as SES and parental time spent with the child were considered [43]. The authors cautioned that higher weight students may be labeled as lower achievers, as weight is a more obvious marker than sociodemographic characteristics.

Adolescents who perceive themselves as overweight may be at risk of internalizing weight stigma. Despite the high prevalence of obesity, weight stigma continues to be problematic. Stereotypes that individuals living with obesity are lazy, unintelligent, or lack willpower contribute to stigma and discrimination [39]. Internalized stigma, or self-stigma, occurs when individuals apply negative stereotypes to themselves and believe that the stigma is deserved [40], leading to low self-esteem and psychological distress [39]. Research indicates that bias toward individuals with overweight and obesity persists in health care, employment, and home settings [9]. Education, however, has received less research attention, particularly at the secondary school level. It is plausible that adolescents who perceive themselves as overweight have lower self-concepts related to internalized weight stigma, which in turn, contributes to poorer academic engagement and performance. That is, students who feel their weight is “about right” may be more likely to succeed because they have not internalized negative stereotypes.

Students with underweight perceptions also reported lower grades than those with “about right” perceptions. Interestingly, the effect of underweight perceptions was more consistent than overweight perceptions in predicting lower grades in both females and males, and across Math and English/French grades. To our knowledge, no previous study has examined underweight perceptions in relation to academic performance. While less studied, underweight perceptions have been associated with depressive and anxiety symptoms in males [21, 44, 45] and suicidality and lower health-related quality of life in all youth [25, 38]. Based on this result, it is plausible that links between weight perception and academic performance relate more to deviations from the social norm or sociocultural body ideals than to weight stigma. Results are consistent with sociocultural body ideals of thinness for women and muscularity for men, with more males reporting underweight perceptions than females. About one-fifth of males and one-tenth of females reported underweight perceptions; yet only 1.5 and 2.1% of female and males had BMIs classified as underweight, respectively. Another plausible explanation is that youth with underweight perceptions were experiencing weight restrictions (e.g., due to food insecurity) and/or had lost weight, while “normal-weight” by BMI, which in turn contributed to reduced ability to perform in school. Results highlight the importance of including weight perceptions across the spectrum in future research.

Interestingly, students that did not report their body weight or weight perception tended to have the lowest likelihood of achieving higher grades, comparable to the odds for obesity BMI classification. Far more males and females with missing BMI data reported perceptions of overweight than their peers with “normal-weight” BMIs. Adolescents are less likely to report their weight as BMI increases and if they have poor body image [32, 46]. Missing self-reported weight status and perceptions may be influenced by an awareness of societal norms of thinness/muscularity ideals and weight bias attitudes [46, 47]. Hence, if adolescents with larger body sizes did not report their weight due to concerns of judgment by others, results lend support to the theory that negative perceptions regarding body weights outside of “about right” contribute to lower academic performance.

Future research should explore both internalized and externalized weight bias, and associated lower self-concept and mental distress, as possible mechanisms explaining links between higher weight status, and perceptions of overweight and underweight, with academic achievement. Upstream strategies targeting the negative connotations of varying body sizes may prove valuable, to prevent the adverse psychosocial outcomes associated with perceptions of being overweight or underweight. Enhanced efforts to prevent weight-based bullying and promote weight acceptance are also advised. Previous research suggests bullying victimization predicts changes from perceptions of being at “about the right weight” to underweight and overweight perceptions among youth [48].

### Limitations

Several limitations require consideration. First, while the large sample supports generalizability, the COMPASS study was not designed to be representative. Second, cross-sectional data was used to explore relationships. Future longitudinal analysis of COMPASS data will assist in establishing temporality and testing potential mechanistic contributors (e.g., mental health, self-concept, bullying victimization, school connectedness). Third, the use of self-reported data carries risks of recall and social desirability bias. For instance, lower achieving students may over report their achievement. However, a review of 37 independent samples found strong response validity of self-reported grades in high school students [49]. Similarly, weight status was based on student-reported height and weight, and as such, results likely reflect greater concordance between weight perception and weight status than exists. However, time and cost constraints preclude the feasibility of obtaining objective height and weight measures, not to mention the potential harm of weight measurements in a school-based study. Also, a strong correlation between measured and self-reported BMI has been shown in youth [30, 50]. Fourth, the reference point that youth used to answer the weight perception question is not entirely clear. That is, it is not known whether respondents were comparing their weight to their ideal body, their peers, a medical standard, or some other alternative. For instance, responses of ‘about the right weight’ may indicate weight satisfaction rather than youths’ perception of how their weight compared to an external reference point. Lastly, as discussed, this study did not assess the contribution of self-esteem or mental health. Mental health may confound the relationship between weight status or perception and academic achievement. Several studies have linked poor mental health with lower grades [37], and a recent intervention promoting positive mental health and wellbeing significantly improved academic grades in participating schools [51]. Future studies, using longitudinal designs, standardized test grades, and including mental health, self-concept, and stigma measures should be considered.

## Conclusion

Results support the existence of an achievement gap by both weight status and weight perception. This study has widespread implications with over 2 million adolescents at risk of having overweight or obesity in Canada [1], and over 40% of youth reporting weight perceptions other than “about right”. Research is needed to further examine the mechanisms underlying these associations. Academic achievement sets a lifelong trajectory of health and wellbeing. Lower academic achievement is linked to increased rates of unemployment, poverty, criminality, and negative future health outcomes [6, 7, 26, 52]. The obesity achievement gap has been suggested as an early contributor to later SES disparities found by weight status [53]. The present study contributes to a body of research that encourages the consideration of both overweight and underweight perceptions and their potential impact on adolescent emotional and physical health. Upstream strategies to prevent negative connotations associated with body sizes divergent from “about right” and to promote weight acceptance merit consideration. Further exploration is necessary to inform policies and interventions that foster a learning environment that will enable all youth to thrive.

## Data Availability

COMPASS study data is available upon request through completion and approval of an online form: https://uwaterloo.ca/compass-system/information-researchers/data-usage-application. The datasets used during the current study are available from the corresponding author on reasonable request.
